# TCM-Derived Small Molecules Targeting Metabolic Vulnerabilities in NSCLC: Ferroptosis-Centered Mechanisms and Emerging Cuproptosis- and Disulfidptosis-Related Vulnerabilities

**DOI:** 10.3390/ph19071026

**Published:** 2026-06-30

**Authors:** Haiyi Zhang, Li Wang, Liang Liu, Yicheng Zhao, Runze Li

**Affiliations:** 1 Chinese Medicine Guangdong Laboratory, Guangzhou University of Chinese Medicine, Zhuhai 519000, China; zzhanghaiyi@163.com (H.Z.); liwang1006@126.com (L.W.); lliu@gzucm.edu.cn (L.L.); 2 State Key Laboratory of Traditional Chinese Medicine Syndrome, Guangdong Provincial Hospital of Chinese Medicine, Guangzhou University of Chinese Medicine, Guangzhou 510000, China

**Keywords:** non-small cell lung cancer, traditional Chinese medicine, ferroptosis, cuproptosis, disulfidptosis

## Abstract

Non-small cell lung cancer (NSCLC) remains the leading cause of cancer-related mortality worldwide and is characterized by therapeutic resistance, metabolic plasticity, and immune evasion. Accumulating evidence indicates that metabolic reprogramming not only supports tumor growth but also creates exploitable vulnerabilities linked to regulated cell death. Traditional Chinese medicine (TCM)-derived small molecules have attracted increasing attention owing to their structural diversity, multitarget properties, and broad pharmacological activities. In this review, we summarize recent advances in TCM-derived compounds targeting metabolism-associated regulated cell death in NSCLC, with a primary focus on ferroptosis and a cautious discussion of emerging cuproptosis- and disulfidptosis-related mechanisms. Ferroptosis has been extensively investigated in this context, with natural compounds shown to induce cell death through coordinated regulation of cystine transport, glutathione metabolism, GPX4 activity, iron homeostasis, and lipid peroxidation. In parallel, emerging studies suggest that certain natural products may influence copper-dependent cell death pathways and metabolic states associated with disulfide stress. These processes are closely linked to distinct metabolic features of NSCLC, including lipid dependency, copper homeostasis, and glucose utilization. Finally, we discuss major challenges for clinical translation, including poor bioavailability, off-target toxicity, insufficient biomarker stratification, and limited high-quality evidence, and highlight emerging strategies such as nanodelivery systems, structural optimization, and targeted protein degradation approaches. Overall, TCM-derived small molecules represent a promising source of metabolism-targeted therapeutics and provide a foundation for further exploration of regulated cell death in NSCLC. Current evidence is strongest for ferroptosis induction, whereas cuproptosis- and disulfidptosis-related mechanisms remain emerging areas that require further experimental validation in NSCLC models.

## 1. Introduction

Lung cancer is among the leading causes of cancer incidence and mortality worldwide and poses a major global public health challenge [1]. In China, lung cancer ranked first in terms of both cancer incidence and cancer-related mortality in 2022. According to estimates released by the National Cancer Center of China, approximately 1.06 million new lung cancer cases and 0.73 million lung cancer-related deaths occurred in China in 2022 [2]. Non-small cell lung cancer is the predominant histological subtype, accounting for approximately 85% of all lung cancer cases [3]. Over the past decade, the therapeutic landscape of NSCLC has substantially changed with the introduction of molecularly targeted therapies and immune checkpoint inhibitors (ICIs), in addition to conventional chemotherapy and radiotherapy [4]. Despite these advances, the overall 5-year survival rate of patients with lung cancer remains approximately 20% in China [5]. These poor outcomes are largely attributed to therapy resistance, disease recurrence and metastasis, and treatment-related toxicity [6]. Therefore, elucidating the molecular mechanisms underlying NSCLC progression and identifying novel mechanism-based therapeutic strategies have become urgent priorities in current cancer research.

Regulated cell death (RCD) is a fundamental biological process that maintains tissue homeostasis and orchestrates cellular responses to stress [7]. Dysregulation of cell death pathways is closely associated with tumorigenesis, therapeutic resistance, and disease progression [8]. For many years, anticancer strategies have focused primarily on caspase-dependent apoptosis. However, accumulating evidence indicates that NSCLC cells can evade apoptosis through genetic and epigenetic alterations, activation of pro-survival signaling pathways, and metabolic adaptation [9]. This has stimulated increasing interest in nonapoptotic forms of RCD that may help overcome classical apoptosis resistance [10]. Notably, growing evidence suggests that cell death pathways are tightly connected with cellular metabolism [11]. Metabolic reprogramming in cancer cells not only supports biosynthetic and energetic demands but also influences their susceptibility to distinct death signals [12,13]. Therefore, targeting metabolism-associated vulnerabilities has emerged as a promising therapeutic strategy in NSCLC.

Among these emerging nonapoptotic death programs, ferroptosis, cuproptosis, and disulfidptosis have attracted increasing attention because of their close association with metabolic imbalance [14]. Ferroptosis is characterized by iron-dependent lipid peroxidation, cuproptosis by copper-induced aggregation of lipoylated mitochondrial proteins, and disulfidptosis by disulfide stress under conditions of glucose deprivation [15]. As these pathways are closely linked to the metabolic dependencies of tumor cells and operate largely independently of classical apoptotic mechanisms, elucidating their regulatory networks may provide new opportunities to overcome apoptosis resistance in NSCLC [16]. However, clinically applicable agents capable of selectively inducing these forms of cell death remain limited. Representative experimental inducers, such as erastin, RSL3, and elesclomol, have provided valuable mechanistic insights; however, their clinical translation is limited by poor pharmacokinetic properties, limited solubility, and potential off-target toxicity [17].

Compared with many single-target synthetic compounds, traditional Chinese medicine (TCM)-derived small molecules often exhibit multitarget and multipathway regulatory properties, which may be advantageous for modulating metabolic reprogramming in NSCLC [18]. Increasing evidence suggests that several TCM-derived monomers can regulate metabolic signaling and induce ferroptosis in NSCLC models, whereas their links to cuproptosis- and disulfidptosis-related vulnerabilities remain emerging and require further validation [19]. In preclinical studies, these compounds have demonstrated antitumor activity and the potential to overcome therapeutic resistance [20].

To ensure the relevance of the included information, we searched PubMed, Web of Science, Scopus, and Google Scholar using combinations of keywords including “traditional Chinese medicine,” “TCM-derived small molecules,” “NSCLC,” “metabolic reprogramming,” “regulated cell death,” “ferroptosis,” “cuproptosis,” and “disulfidptosis.” Studies were included when they focused on TCM-derived small molecules or natural compounds with mechanistic evidence related to metabolism-associated regulated cell death in NSCLC or closely relevant cancer models. Studies were excluded if they focused only on crude herbal formulas without identifiable active compounds, lacked mechanistic relevance to metabolism-associated cell death, or were unrelated to cancer biology.

Accordingly, this review summarizes recent advances in TCM-derived small molecules that modulate metabolism-associated cell death in NSCLC, with an emphasis on ferroptosis and emerging cuproptosis- and disulfidptosis-related vulnerabilities. We further discuss their molecular mechanisms, possible structure–activity relationships, and future clinical prospects, with the aim of providing insights into the development of metabolism-based therapeutic strategies for NSCLC. By integrating evidence from cancer metabolism, regulated cell death, TCM pharmacology, and natural product-based drug discovery, this review may be useful for researchers and clinicians interested in mechanism-based anticancer strategies and translational oncology.

To provide a concise overview of the most relevant compounds discussed in this review, representative TCM-derived small molecules selected from Supplementary Table S1 are summarized in Table 1, together with their chemical structures and associated metabolism-related cell death modalities. Detailed information, including additional compounds, molecular targets, mechanisms, and supporting references, is provided in Supplementary Table S1.

## 2. Ferroptosis: Iron-Dependent Lipid Peroxidation

Ferroptosis is a form of regulated cell death first described in 2012 by Dixon and colleagues and is characterized by the lethal accumulation of iron-dependent lipid peroxides on cellular membranes [21,22]. In terms of morphology, genetics, and biochemistry, ferroptosis is distinct from apoptosis, necroptosis, and other classical forms of cell death [23]. It lacks hallmark features of apoptosis, such as chromatin condensation, nuclear fragmentation, and caspase-3 activation. Instead, its characteristic ultrastructural alterations are primarily observed in mitochondria, including reduced mitochondrial volume, increased membrane density, and loss of mitochondrial cristae [24]. Pharmacological studies have shown that ferroptosis can be inhibited by iron chelators and lipophilic antioxidants but is not prevented by classical inhibitors of apoptosis or necrosis [25].

In NSCLC, increasing evidence suggests that ferroptosis represents a critical factor associated with metabolic reprogramming and redox imbalance [26]. Notably, tumor cells that develop resistance to chemotherapy or targeted therapy often exhibit enhanced dependence on antioxidant defense systems, thereby rendering them potentially susceptible to ferroptosis induction [27]. Given its unique mechanism and metabolic dependency, ferroptosis has emerged as a promising strategy for eliminating therapy-resistant tumor cells. In this context, this review focuses primarily on compounds that are directly derived from traditional Chinese medicinal materials or their bioactive monomers that have been historically used or systematically studied within the framework of Chinese medicine.

### 2.1. Mechanisms of Ferroptosis in NSCLC

#### 2.1.1. Canonical System Xc^−^/GSH/GPX4 Axis

Glutathione peroxidase 4 (GPX4) is widely recognized as a central regulator of the ferroptosis defense system. As a selenoenzyme with high substrate specificity, GPX4 utilizes reduced glutathione (GSH) as an essential cofactor to reduce toxic phospholipid hydroperoxides (PL-OOH) into nontoxic lipid alcohols, thereby preventing the propagation of lipid peroxidation chain reactions [28]. The antioxidant activity of GPX4 critically depends on the availability of intracellular GSH. Cysteine, the rate-limiting precursor for GSH synthesis, is primarily supplied through the uptake of extracellular cystine via the membrane transporter System Xc^−^, which consists of the catalytic subunit SLC7A11 and the regulatory subunit SLC3A2 [29].

In NSCLC, hyperactivation of the system Xc^−^/GSH/GPX4 axis represents a key mechanism of ferroptosis resistance. Clinical and genomic evidence indicates that loss-of-function mutations in KEAP1 or activating alterations in NRF2 frequently drive the transcriptional upregulation of SLC7A11, thereby increasing the antioxidant capacity and enabling tumor cells to adapt to metabolic stress [30,31]. This metabolic reprogramming not only supports tumor growth but also contributes to therapeutic resistance. Consequently, NSCLC cells frequently rely on this antioxidant axis to maintain redox homeostasis, which may represent therapeutic vulnerability. Consistently, inhibition of cystine uptake or direct targeting of GPX4 has been shown to induce lipid peroxidation and ferroptotic cell death in NSCLC models [32].

#### 2.1.2. Iron Metabolism and the Fenton Reaction

The initiation of ferroptosis critically depends on the pathological expansion of the intracellular labile iron pool (LIP). Iron serves not only as an essential cofactor for numerous metabolic enzymes but also as a key catalyst driving lipid peroxidation chain reactions [33]. Under physiological conditions, circulating ferric iron (Fe^3+^) binds to transferrin and is internalized into cells via transferrin receptor 1 (TFR1)-mediated endocytosis [34]. To prevent toxicity from excess free iron, intracellular iron is tightly regulated and stored in ferritin complexes. During ferroptosis, this homeostatic balance is disrupted. Nuclear receptor coactivator 4 (NCOA4) functions as a selective autophagy receptor that mediates the lysosomal degradation of ferritin, a process termed ferritinophagy [35]. This process releases substantial amounts of ferrous iron (Fe^2+^) into the cytosol, leading to the expansion of LIP. The accumulated Fe^2+^ subsequently catalyzes the Fenton reaction, converting hydrogen peroxide into highly reactive hydroxyl radicals, which directly attack polyunsaturated fatty acids in cellular membranes and initiate lipid peroxidation [21]. In NSCLC, tumor cells frequently exhibit increased iron demand to sustain rapid proliferation, a phenomenon often referred to as “iron addiction.” This state is commonly associated with the upregulation of TFR1 and the suppression of iron export pathways. Such metabolic features not only support tumor growth but also may sensitize NSCLC cells to ferroptosis under conditions of iron overload or disrupted iron homeostasis. Accordingly, strategies that promote ferritinophagy or increase intracellular free iron levels have been suggested to increase ferroptosis in NSCLC models [36].

#### 2.1.3. Lipid Remodeling and Peroxidation in Ferroptosis

Lipid peroxidation represents the terminal execution step of ferroptosis, although not all lipid species are equally susceptible to oxidation. Ferroptosis preferentially targets polyunsaturated fatty acids (PUFAs) within membrane phospholipids, particularly arachidonic acid and adrenic acid. Because PUFAs contain bisallylic hydrogen atoms that are highly susceptible to abstraction by reactive oxygen species, they provide the chemical basis for ferroptotic lipid peroxidation [37]. This process is tightly regulated by lipid-remodeling enzymes, among which long-chain acyl-CoA synthetase 4 (ACSL4) and lysophosphatidylcholine acyltransferase 3 (LPCAT3) play central roles. ACSL4 catalyzes the activation of free PUFAs into PUFA-CoA species, whereas LPCAT3 incorporates these activated fatty acids into membrane phospholipids, leading to the formation of oxidation-prone PUFA-containing phosphatidylethanolamines (PE-PUFAs). Accordingly, ACSL4 is widely recognized as a key determinant of ferroptosis sensitivity by expanding the pool of oxidizable PUFA-containing phospholipids [38]. Once enriched in membrane phospholipids, these PUFA species undergo extensive peroxidation catalyzed by lipoxygenases or cytochrome P450 oxidoreductase, resulting in the accumulation of lethal lipid peroxides [39]. In NSCLC, emerging evidence suggests that lipid remodeling plays an important role in modulating ferroptosis susceptibility. ACSL4 has been proposed as a potential biomarker for predicting the responsiveness to ferroptosis-inducing therapies. Notably, some drug-resistant NSCLC cells exhibit reduced PUFA incorporation into membrane phospholipids, which has been associated with decreased ACSL4 expression and a ferroptosis-resistant phenotype [40]. This adaptive lipid remodeling may represent a metabolic mechanism through which tumor cells evade ferroptotic cell death. Accordingly, pharmacological strategies aimed at restoring ACSL4 activity or promoting lipid peroxidation may help sensitize resistant NSCLC cells to ferroptosis [41].

#### 2.1.4. Noncanonical Defense Systems

GPX4 has long been regarded as the central component of the ferroptosis defense system. However, accumulating evidence indicates that some tumor cells can remain viable even in the absence of GPX4, suggesting the existence of noncanonical anti-ferroptosis mechanisms that operate in parallel with the GPX4 pathway [42]. Among these pathways, the FSP1–CoQ10–NAD(P)H pathway is among the best characterized independent defense systems [42]. Ferroptosis suppressor protein 1 (FSP1), localized at the plasma membrane, functions as an oxidoreductase that utilizes NAD(P)H to reduce ubiquinone (CoQ10) to ubiquinol (CoQ10H2) [43]. Ubiquinol acts as a potent lipophilic radical-trapping antioxidant that terminates lipid peroxidation chain reactions at the membrane, thereby compensating for the loss of GPX4 activity [44]. In addition to membrane-associated defense, mitochondrial protection is mediated by dihydroorotate dehydrogenase (DHODH), which reduces CoQ to CoQH2 while catalyzing pyrimidine synthesis, thereby limiting lipid peroxidation within mitochondria [45]. Furthermore, the cytosolic GCH1–BH4 axis generates tetrahydrobiopterin (BH4), which functions not only as a metabolic cofactor but also as a lipid-protective antioxidant that prevents the oxidation of PUFA-containing phospholipids [46]. Collectively, these pathways constitute a parallel ferroptosis defense network that complements or compensates for GPX4-dependent antioxidant systems.

In NSCLC, emerging evidence suggests that FSP1-mediated ferroptosis defense may contribute to reduced sensitivity to GPX4 inhibition and ferroptosis-inducing therapies. Lung cancer cells or tumors with elevated FSP1 expression have been reported to exhibit ferroptosis resistance and may benefit from FSP1-targeted strategies [47]. This redundancy in ferroptosis defense mechanisms highlights the potential limitations of single-target strategies. Accordingly, cotargeting canonical and noncanonical ferroptosis defense systems, such as GPX4 and FSP1 or DHODH, may represent a promising approach to overcome ferroptosis resistance in NSCLC.

### 2.2. TCM-Derived Small Molecules That Target Ferroptosis

#### 2.2.1. Natural Products Targeting the SLC7A11/GSH/GPX4 Antioxidant Defense Axis

Disruption of the SLC7A11/GSH/GPX4 antioxidant defense system represents the most consistently supported mechanism by which natural products induce ferroptosis in non-small cell lung cancer (NSCLC), as this pathway is central to the detoxification of lipid peroxides and the maintenance of redox homeostasis [21,48]. Among the currently available studies, β-elemene appears to be among the strongest examples in NSCLC because it induces ferroptotic cell death through TFEB-mediated lysosomal degradation of GPX4, thereby directly impairing the GPX4-centered antioxidant defense machinery [49]. Importantly, the significance of β-elemene is not limited to mechanistic observations in cultured cells; it has also been associated with enhanced therapeutic responses in EGFR-targeted settings, suggesting that ferroptosis induction may contribute to its potential translational value in NSCLC treatment [50]. In addition to β-elemene, periplocin provides another important line of evidence, as recent work has shown that it potentiates ferroptotic cell death in NSCLC by promoting NRF2 degradation, thereby weakening antioxidant defense programs upstream of GPX4 and SLC7A11 [51]. This finding is particularly noteworthy because it suggests that natural products may trigger ferroptosis not only by acting on GPX4 directly but also by destabilizing the transcriptional network that sustains ferroptosis resistance. By comparison, ophiopogonin B can be mentioned as a supplementary example, since it has also been reported to induce ferroptosis-related death in NSCLC cells, although its mechanistic resolution and translational depth remain less comprehensive than those reported for β-elemene and periplocin [52]. Taken together, the current evidence indicates that the SLC7A11/GSH/GPX4 axis is the best-supported ferroptosis-related target of natural products in NSCLC, with β-elemene and periplocin representing the most convincing examples at present.

#### 2.2.2. Targeting Iron Homeostasis and Fenton Chemistry

Iron homeostasis is another important route through which natural products promote ferroptosis in NSCLC because the expansion of the intracellular labile iron pool facilitates lipid peroxidation and amplifies oxidative damage through iron-dependent redox reactions [53]. Among traditional Chinese medicine-derived compounds, artemisinin derivatives are representative iron-responsive agents. In NSCLC models, artemisinin derivatives, including dihydroartemisinin (DHA), have been shown to inhibit tumor cell growth through ROS-dependent apoptosis/ferroptosis, supporting the view that this class of compounds can engage in ferroptosis-related death programs in lung cancer [54]. Mechanistically, the ferroptosis-promoting activity of artemisinin compounds is closely linked to their iron-reactive endoperoxide bridge, which can be activated by intracellular iron to generate highly reactive radical species and thereby intensify oxidative injury [55]. In addition, evidence from other cancer models has indicated that DHA or artesunate may further increase ferroptotic vulnerability by promoting ferritinophagy and increasing intracellular free iron levels, although the full NSCLC-specific validation of the NCOA4-dependent branch remains to be performed [54,56].

In addition to mobilizing intracellular iron stores, some natural products may also promote ferroptosis by enhancing iron uptake. Cucurbitacin B is currently among the clearest examples in NSCLC, as it has been reported to target STAT3 and induce ferroptosis in non-small cell lung cancer, thereby linking STAT3 inhibition to ferroptosis-associated iron overload and lipid peroxidation [57]. By comparison, shikonin is better discussed as a supportive rather than central example in lung cancer, since stronger direct evidence is currently available concerning small cell lung cancer, where shikonin suppresses tumor growth by inducing ATF3-dependent ferroptosis [58]. Overall, the current evidence suggests that natural products can promote ferroptosis through two interrelated iron-centered routes: mobilization of intracellular iron stores and enhancement of iron-dependent oxidative injury, with artemisinin derivatives and cucurbitacin B representing the most relevant examples for this subsection.

#### 2.2.3. Targeting NRF2/KEAP1 Signaling and Lipid Remodeling

Targeting the NRF2/KEAP1 signaling pathway represents a potential strategy to modulate ferroptosis sensitivity in non-small cell lung cancer (NSCLC), particularly in tumors characterized by elevated antioxidant capacity [59]. NRF2 is a master regulator of cellular redox homeostasis, and its activation promotes the transcription of multiple ferroptosis-suppressive genes, including SLC7A11, GPX4, and GCLM, thereby establishing a robust antiferroptotic defense program [60]. Among natural compounds, brusatol has been widely investigated as an inhibitor of NRF2 signaling. Mechanistic studies have indicated that brusatol promotes NRF2 ubiquitination and proteasomal degradation, resulting in rapid suppression of NRF2-dependent antioxidant gene expression [61]. Although its ferroptosis-specific role in NSCLC remains to be fully established, available evidence suggests that inhibition of NRF2 by brusatol can sensitize lung cancer cells to oxidative stress and may lower the threshold for ferroptotic cell death.

Similarly, the activity of trigonelline, a plant-derived alkaloid, interferes with NRF2 signaling by blocking its nuclear translocation and downregulating the expression of downstream antioxidant genes such as GCLM, thereby reducing intracellular glutathione synthesis [62]. In lung cancer cell models such as A549 cells, this effect contributes to enhanced oxidative stress and promotes a cellular environment favorable to lipid peroxidation, although direct evidence linking trigonelline to canonical ferroptosis pathways in NSCLC is still limited. In addition, oridonin has been shown to suppress NRF2-associated signaling and promote the accumulation of reactive oxygen species in cancer cells, including lung cancer cells [63]. Emerging studies suggest that this redox imbalance may be associated with ferroptosis-like cell death; however, the specific involvement of the ferroptotic machinery requires further validation in NSCLC systems.

In addition to redox regulation, lipid metabolism remodeling represents another important mechanism through which natural products may increase ferroptosis susceptibility. The abundance of polyunsaturated fatty acid (PUFA)-containing phospholipids in cellular membranes is a key determinant of ferroptotic sensitivity, as these lipids serve as substrates for peroxidation [64]. In this context, polyphyllins (such as polyphyllin I, II, and VI) have been reported to regulate lipid metabolism in cancer cells by upregulating the expression of ACSL4, a key enzyme that catalyzes the activation and incorporation of PUFAs into membrane phospholipids [65]. Increased ACSL4 expression expands the pool of oxidizable lipids and, together with the expression of lipid-remodeling enzymes such as LPCAT3, increases membrane susceptibility to peroxidation under oxidative stress [66]. Certain natural products derived from marine organisms have also been reported to disrupt the balance between ACSL4 and GPX4, thereby promoting lipid peroxide accumulation and ferroptosis-related cell death [67]. Although evidence in NSCLC remains limited, these findings support the broader concept that lipid metabolic reprogramming is an important regulatory layer of ferroptosis.

Overall, in contrast to direct inhibition of GPX4, targeting NRF2 signaling and lipid remodeling primarily occurs at the transcriptional and metabolic levels, thereby reshaping ferroptosis susceptibility rather than directly triggering cell death. Natural compounds such as brusatol and trigonelline may weaken NRF2-dependent antioxidant defenses, whereas polyphyllins increase the availability of oxidizable lipid substrates. This dual regulatory mode provides a mechanistic basis for sensitizing NSCLC cells—especially those with strong antioxidant capacity—to ferroptosis-based therapeutic strategies.

#### 2.2.4. Ferroptosis Induction for Overcoming Drug Resistance

Acquired resistance to chemotherapy and targeted therapies remains a major challenge in the treatment of non-small cell lung cancer (NSCLC). Increasing evidence suggests that resistant tumor cells frequently exhibit enhanced antioxidant capacity, particularly through activation of the SLC7A11/GSH/GPX4 axis, which has been implicated in ferroptosis resistance and may contribute to therapeutic failure [48,68]. Within this framework, a limited number of TCM-derived compounds have been directly validated to target this vulnerability in NSCLC. Among them, β-elemene represents one of the most compelling examples, as it promotes GPX4 degradation through TFEB-mediated lysosomal pathways, thereby disrupting the core antioxidant defense system and sensitizing tumor cells to lipid peroxidation [49]. Similarly, periplocin has been shown to potentiate ferroptotic cell death by inducing NRF2 degradation, which weakens the transcriptional program that maintains redox homeostasis and ferroptosis resistance in NSCLC cells [51].

On the basis of this mechanism, ferroptosis induction may also increase the efficacy of existing therapies. For example, cotreatment with betulin and gefitinib has been reported to induce ferroptosis and improve antitumor responses in EGFR-targeted settings, suggesting that ferroptosis activation can help restore drug sensitivity [69]. In addition to direct induction and combination strategies, some compounds may modulate ferroptosis-related susceptibility. Cucurbitacin B, for example, has been reported to induce ferroptosis in NSCLC by targeting STAT3 and promoting oxidative stress, thereby linking oncogenic signaling pathways to ferroptosis sensitivity [57].

**Figure 1:**
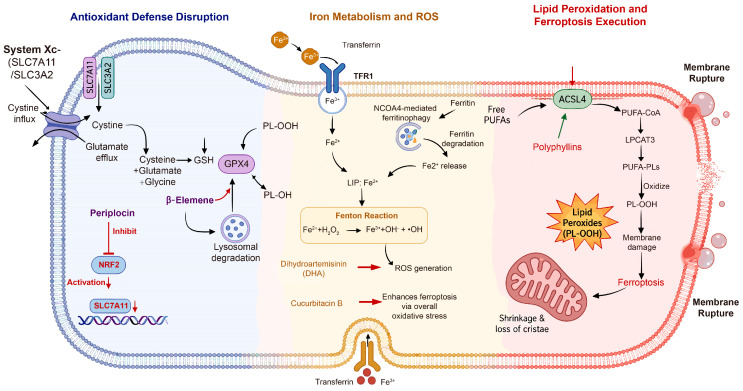
TCM-derived small molecules induce ferroptosis in NSCLC by targeting antioxidant defense, iron metabolism, and lipid peroxidation. Schematic representation of the mechanisms by which TCM-derived small molecules induce ferroptosis in NSCLC cells. Disruption of antioxidant defense via inhibition of the system Xc–GSH–GPX4 axis reduces cellular redox capacity. Concurrently, the modulation of iron metabolism increases the labile iron pool and promotes ROS generation through the Fenton reaction. Enhanced lipid peroxidation, mediated by enzymes such as ACSL4 and LPCAT3, leads to the accumulation of toxic lipid peroxides and ultimately to ferroptotic cell death. Red and green arrows or lines indicate the regulatory effects of TCM-derived small molecules on specific targets or processes, whereas black arrows indicate endogenous molecular transport, biochemical reactions, or pathway progression. Blunt-ended lines indicate inhibitory effects.

Current evidence indicates that ferroptosis induction represents a promising strategy for overcoming drug resistance in NSCLC; however, the number of well-validated TCM-derived ferroptosis inducers remains limited, and many compounds act primarily by reshaping redox homeostasis rather than directly triggering ferroptosis. Further studies are therefore needed to establish more definitive mechanistic links and to translate these findings into clinically applicable strategies. The major ferroptosis-regulatory mechanisms targeted by TCM-derived small molecules in NSCLC, including antioxidant defense disruption, iron metabolic remodeling, and lipid peroxidation amplification, are summarized in Figure 1.

## 3. Copper-Dependent Cell Death and Cuproptosis-Related Vulnerability

### 3.1. Mechanistic Basis of Cuproptosis

Copper-induced cytotoxicity has traditionally been attributed to nonspecific oxidative stress, often involving Fenton-like reactions. This view was substantially revised by a seminal study from Tsvetkov and colleagues in 2022, which defined a distinct form of copper-dependent regulated cell death termed cuproptosis [70]. Mechanistically, cuproptosis differs from apoptosis, ferroptosis, and necroptosis and is not prevented by inhibitors targeting these pathways [71]. Its core mechanism involves the direct binding of intracellular copper to lipoylated proteins of the mitochondrial tricarboxylic acid (TCA) cycle, leading to aberrant protein aggregation and the subsequent loss of iron–sulfur (Fe–S) cluster proteins. This process induces proteotoxic stress and mitochondrial dysfunction, which are considered defining features of cuproptosis, rather than nonspecific reactive oxygen species accumulation [72]. However, it is important to note that copper-dependent cytotoxicity should not be equated with canonical cuproptosis unless these defining molecular features are demonstrated. In the context of non-small cell lung cancer (NSCLC), current evidence remains limited, and many studies have described copper-induced cell death without fully validating the hallmark mechanisms of cuproptosis. Therefore, a careful distinction between bona fide cuproptosis and general copper-mediated toxicity is essential when experimental findings are interpreted and strategies are evaluated.

#### 3.1.1. Copper Homeostasis and Cellular Copper Handling

Copper serves as an essential catalytic cofactor for key enzymes such as cytochrome c oxidase (COX) and superoxide dismutase 1 (SOD1) and plays a critical role in mitochondrial respiration and redox homeostasis [73]. However, free copper ions are highly redox-active and potentially toxic. Without strict regulation, they can participate in redox cycling and aberrant interactions with macromolecules [74]. Therefore, NSCLC cells rely on a tightly controlled network of transporters and chaperones to maintain low levels of bioavailable intracellular copper. Disruption of this homeostasis may activate cell death pathways [75]. To support the elevated metabolic demand associated with rapid proliferation and angiogenesis, NSCLC cells often exhibit increased expression of the high-affinity copper transporter CTR1 (also known as SLC31A1) [76]. Following uptake, intracellular Cu^+^ functions not only as a metabolic cofactor but also in signaling pathways such as MAPK/ERK signaling, thereby contributing to tumor progression [77]. Clinical observations further support the clinical relevance of CTR1 in NSCLC, as genetic variation in CTR1 has been associated with severe cisplatin-induced toxicity in patients [78]. While this phenotype may support tumor growth, it may also increase susceptibility to copper-dependent cytotoxicity under conditions of copper overload.

Once Cu^+^ enters the cytosol through CTR1, it is rapidly bound by glutathione (GSH), which acts as an important buffering system to limit copper toxicity [79]. Copper-induced cytotoxicity becomes more pronounced when copper influx exceeds the buffering capacity of intracellular thiols. In addition to GSH, the copper chaperone ATOX1 plays a critical role in intracellular copper trafficking. ATOX1 delivers copper to the secretory pathway and has also been reported to translocate to the nucleus, where it may regulate gene expression associated with cell proliferation [80]. When intracellular copper levels increase, the copper exporters ATP7A and ATP7B relocalize from the Golgi apparatus to the plasma membrane to facilitate copper efflux [81]. Notably, platinum-based chemotherapeutic agents such as cisplatin share components of this transport system, and overexpression of ATP7A/B has been associated with reduced intracellular drug accumulation and therapeutic resistance in NSCLC [82].

Overall, NSCLC cells maintain a dynamic balance between copper uptake, intracellular buffering, and efflux. Disrupting this balance may create a cellular state permissive for copper-dependent cytotoxicity. Copper ionophores, for example, can increase intracellular copper availability and overwhelm cellular buffering systems, thereby promoting copper-induced stress responses that may contribute to cuproptosis under appropriate molecular conditions [83].

#### 3.1.2. FDX1–Lipoylation–TCA Cycle Axis

Mitochondria serve as the central execution site of cuproptosis. Ferredoxin 1 (FDX1) has been identified as a key upstream regulator of this process, acting as a mitochondrial reductase that reduces Cu^2+^ to the more reactive Cu^+^ form, thereby facilitating copper-induced cytotoxicity [84]. In addition to its role in copper redox cycling, the FDX1/LIAS axis is closely linked to mitochondrial protein lipoylation, thereby maintaining the lipoylation status of key enzyme complexes within the tricarboxylic acid (TCA) cycle, including pyruvate dehydrogenase (PDH) and α-ketoglutarate dehydrogenase (α-KGDH) complexes [85]. In the context of cuproptosis, copper does not induce cell death through lipid peroxidation, as observed in ferroptosis. Instead, Cu^+^ selectively binds to lipoylated components of the TCA cycle, particularly dihydrolipoamide S-acetyltransferase (DLAT), leading to abnormal protein aggregation and oligomerization [86].

This aggregation of lipoylated proteins results in the accumulation of insoluble protein complexes, triggering proteotoxic stress and mitochondrial dysfunction. Concurrently, copper overload has been shown to destabilize iron–sulfur (Fe–S) cluster proteins, further impairing mitochondrial respiration and contributing to cell death [87]. Importantly, genetic or pharmacological disruption of FDX1 or protein lipoylation is sufficient to suppress copper-induced cell death, highlighting the essential role of the FDX1–lipoylation axis in mediating cuproptosis [85]. Given that NSCLC cells frequently exhibit metabolic reprogramming and retain active mitochondrial respiration, they may be particularly sensitive to perturbations in lipoylated enzyme complexes and mitochondrial proteostasis. However, direct disease-specific validation in NSCLC remains relatively limited, and further validation in disease-specific models is needed [88,89].

These findings reveal that the FDX1-dependent lipoylation axis is vulnerable to mitochondrial metabolism and can be exploited by copper overload. Pharmacological strategies that increase intracellular copper availability may promote copper-dependent protein aggregation and proteotoxic stress, thereby facilitating cuproptosis under appropriate molecular conditions.

### 3.2. TCM-Derived Small Molecules Modulate Copper-Dependent Cell Death in NSCLC

#### 3.2.1. Celastrol as a Representative Compound

Among TCM-derived small molecules, celastrol currently represents one of the strongest examples linking copper uptake to cuproptosis-associated cell death in NSCLC. A recent study in A549 and HCC4006 cells revealed that celastrol inhibited proliferation, migration, and invasion and that its cytotoxicity was rescued more effectively by the copper chelator tetrathiomolybdate than by inhibitors of other regulated cell death pathways, supporting a copper-dependent mechanism [90]. Mechanistically, celastrol upregulates the expression of the copper transporter SLC31A1/CTR1 through SRF-dependent transcriptional control, thereby increasing intracellular copper accumulation [90]. In the same study, celastrol treatment was associated with decreased GSH levels; DLAT oligomerization; increased HSP70 expression; and the loss of iron–sulfur cluster-related proteins, including FDX1, SDHB, and POLD1, together with reduced mitochondrial membrane potential and ATP production. These changes overlap substantially with the molecular features originally used to define cuproptosis [70].

This evidence places celastrol apart from many other natural compounds that have been linked only to nonspecific copper toxicity or oxidative stress. Although celastrol is a pleiotropic natural product and has previously been reported to exert antitumor effects through ROS accumulation, STAT3 inhibition, and apoptosis-related pathways in NSCLC, the recently described SRF/SLC31A1-driven copper accumulation and cuproptosis-associated molecular changes provide a more direct mechanistic connection to copper-dependent cell death [91]. Therefore, celastrol may be regarded as a representative TCM-derived compound that affects cuproptosis-related vulnerability in NSCLC.

#### 3.2.2. Other Compounds and Indirect Modulation of Cuproptosis-Related Pathways

Direct evidence linking natural products to copper homeostasis and cuproptosis in NSCLC remains limited. However, available studies suggest that natural compounds may influence this process through two interconnected routes: direct modulation of copper transport or copper chaperone networks and indirect alteration of cellular states that determine susceptibility to copper-induced death, particularly mitochondrial metabolism and proteostasis [92,93,94].

Among the currently reported compounds, curcumin represents the clearest example of a natural product that directly modulates copper homeostasis in NSCLC [92]. A recent study revealed that curcumin binds to the copper chaperone ATOX1, suppresses intracellular copper accumulation, and inhibits ATOX1-mediated copper signaling in NSCLC cells [92]. Functionally, this effect was associated with reduced tumor cell growth, indicating that interference with the copper trafficking machinery may constitute one important component of the anticancer activity of selected natural compounds in lung cancer. These findings are mechanistically informative because they suggest that natural products in NSCLC can act not only on general oxidative stress pathways but also on specific nodes of copper handling.

In addition to directly affecting copper homeostasis, some natural compounds may influence cuproptosis-related vulnerability through the remodeling of mitochondrial metabolism. Cuproptosis is tightly linked to mitochondrial activity because excess copper binds lipoylated components of the tricarboxylic acid cycle, promotes mitochondrial protein aggregation, and destabilizes Fe–S cluster proteins [70]. In this context, triptolide provides a representative example in NSCLC. Previous work has shown that triptolide impairs mitochondrial bioenergetics in NSCLC cells in a p53-dependent manner, partly through the dysregulation of SIRT3 and the reduced activity of respiratory chain complexes I and II [93]. Although this study did not directly examine cuproptosis, it suggests that natural compounds capable of rewiring mitochondrial metabolism may alter the metabolic background that determines copper sensitivity.

A second potentially relevant route involves proteostasis. Because copper toxicity is closely associated with mitochondrial protein aggregation and proteotoxic stress, the protein quality-control machinery is likely to influence cellular tolerance to copper-induced injury [95]. In cancer cells, HSP90-centered chaperone networks stabilize multiple oncogenic client proteins and help buffer proteotoxic stress. Natural products such as gambogic acid have been identified as HSP90 inhibitors, and strategies targeting HSP90 have been shown to be relevant in NSCLC [94]. Moreover, combined inhibition of HSP90 and the proteasome has been reported to intensify unfolded protein response signaling and endoplasmic reticulum stress in NSCLC models [95]. These observations support the idea that weakening proteostasis may lower the threshold for copper-triggered proteotoxic damage.

**Figure 2:**
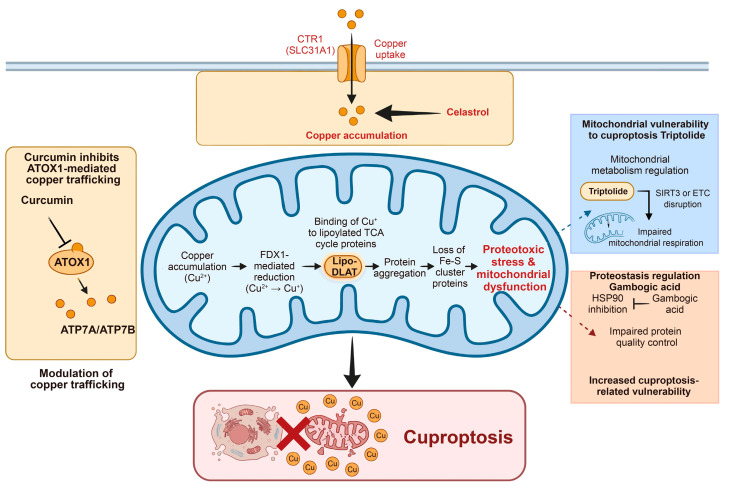
TCM-derived compounds modulate cuproptosis-related vulnerability by regulating copper homeostasis, mitochondrial metabolism, and proteostasis in NSCLC. Schematic illustration of cuproptosis-related mechanisms regulated by TCM-derived compounds in NSCLC cells. Increased intracellular copper accumulation, partly mediated by CTR1/SLC31A1-dependent copper uptake, promotes FDX1-mediated reduction of Cu^2+^ to Cu^+^. Cu^+^ binds to lipoylated TCA-cycle proteins, especially lipoylated DLAT, leading to lipoylated protein aggregation, loss of Fe–S cluster proteins, proteotoxic stress, mitochondrial dysfunction, and cuproptosis. TCM-derived compounds may modulate this process by affecting copper uptake and trafficking, mitochondrial respiration, and proteostasis, thereby altering cellular susceptibility to cuproptosis-related death.

Current evidence suggests that natural compounds in NSCLC may affect cuproptosis-related processes at multiple levels, including copper transport, copper chaperone systems, mitochondrial metabolism, and proteostasis. However, in addition to the evidence concerning celastrol discussed above, most currently available evidence remains indirect. In most cases, these compounds have been shown to perturb pathways that are theoretically relevant to cuproptosis rather than being directly demonstrated to induce or sensitize NSCLC cells to cuproptosis. Further studies are therefore needed to determine whether the modulation of these pathways can be more broadly translated into validated cuproptosis-based therapeutic strategies in NSCLC. Based on these evidence levels, Figure 2 illustrates how selected TCM-derived compounds may modulate cuproptosis-related vulnerability through copper handling, mitochondrial metabolism, and proteostasis, rather than uniformly acting as bona fide cuproptosis inducers.

## 4. Disulfidptosis: A Glucose Deprivation-Associated Form of RCD

### 4.1. Mechanistic Basis and Potential Relevance of Disulfidptosis in NSCLC

Disulfidptosis is a recently characterized form of regulated cell death triggered by disulfide stress under conditions of glucose deprivation, and it is mechanistically distinct from apoptosis, ferroptosis, and other established types of death programs. In a study that first defined this process, glucose starvation in SLC7A11-high cancer cells induced aberrant intracellular disulfide accumulation, extensive disulfide bonding in actin cytoskeleton proteins, and subsequent collapse of the F-actin network, ultimately leading to cell death [15]. Its metabolic basis is closely related to the dual role of SLC7A11/xCT. Although SLC7A11 promotes cystine uptake and supports glutathione synthesis, sustained cystine import also imposes a substantial demand for reducing equivalents, thereby increasing cellular dependence on glucose metabolism and NADPH production [96]. When the glucose supply becomes limited, NADPH depletion compromises cystine reduction and drives intracellular disulfide accumulation, converting the redox advantage associated with SLC7A11 into metabolic vulnerability [15]. A defining downstream feature of disulfidptosis is therefore not lipid peroxidation, as in ferroptosis, but the collapse of the actin cytoskeleton caused by aberrant disulfide bonding. Mechanistic analyses further revealed that regulators of actin dynamics influence cellular susceptibility to this process, with the WAVE regulatory complex acting as a positive determinant and Rac activation further promoting cell death under glucose-starved conditions [97].

This mechanism may be particularly relevant to NSCLC because lung cancer cells frequently exhibit marked metabolic rewiring, including increased reliance on glucose utilization and redox-adaptive pathways. In NSCLC, SLC7A11 overexpression has been shown to promote metabolic reprogramming and tumor progression, supporting the view that a subset of lung cancers may exist in a state of heightened dependence on the SLC7A11–glucose metabolism axis [96]. Consistent with these findings, KEAP1-deficient lung cancer cells, in which NRF2 activation drives SLC7A11 upregulation, display stronger glucose dependency and greater sensitivity to glucose deprivation or GLUT inhibition, indicating that disulfide stress-associated metabolic vulnerability can be therapeutically exposed in lung cancer [98]. These findings suggest that the mechanistic prerequisites for disulfidptosis, including high SLC7A11 activity, dependence on glucose-derived reducing power, and vulnerability to disulfide stress, are biologically plausible in NSCLC, although direct evidence for bona fide disulfidptosis in this setting remains limited. This also provides a mechanistic basis for examining whether compounds that restrict glucose uptake or glycolytic flux may create a permissive context for disulfidptosis in NSCLC [98].

### 4.2. TCM-Derived Compounds Targeting Glucose Metabolism with Potential Relevance to Disulfidptosis in NSCLC

Given that disulfidptosis is initiated when SLC7A11-high cells experience glucose deprivation and fail to maintain the reducing power required to resolve intracellular disulfide stress, interventions that restrict glucose availability or glycolytic flux are mechanistically relevant to this death program [99]. In NSCLC, this connection is particularly plausible because glucose transport and glucose utilization are not only passive metabolic features but also active determinants of tumor growth, therapeutic response, and metabolic adaptation [100,101]. Accordingly, TCM-derived compounds that suppress glucose uptake, reduce glycolytic throughput, or interfere with upstream signaling pathways to control these processes may create a cellular context potentially permissive for disulfidptosis, even when this mode of death has not yet been directly demonstrated in NSCLC for a given agent [98].

#### 4.2.1. TCM-Derived Compounds Targeting Glucose Transporters in NSCLC

Among glucose transport systems, GLUT1 is the most consistently implicated transporter in NSCLC and appears to be especially relevant to metabolically vulnerable subsets. Clinical and experimental studies have shown that GLUT1 is highly expressed in NSCLC, promotes malignant phenotypes, and contributes to therapeutic resistance, whereas pharmacological or genetic inhibition of GLUT1 suppresses tumor growth and sensitizes resistant cells to treatment [102]. These findings are further supported by the findings of subtype-specific metabolic work showing that lung squamous cell carcinoma displays particularly high GLUT1 expression and increased susceptibility to glucose deprivation or GLUT1 inhibition, indicating that the dependence on transporter-mediated glucose uptake can itself represent therapeutically exploitable metabolic liability in NSCLC [103].

At present, however, direct evidence that TCM-derived compounds specifically target glucose transporters in NSCLC and thereby induce disulfidptosis remains limited. A more cautious interpretation of the available literature is that some natural compounds may influence this axis either by downregulating transporter expression or by suppressing upstream signaling pathways that sustain glucose uptake, rather than by being unequivocally validated as disulfidptosis inducers through transporter blockade. In this context, the relevance of transporter-targeting natural products should be understood primarily at the level of metabolic priming: by restricting glucose entry into tumor cells, such agents may reduce NADPH-generating capacity and thereby increase susceptibility to disulfide stress in SLC7A11-high NSCLC cells [104]. These findings also suggest that future studies should test more directly whether natural compounds that impair GLUT1-dependent glucose uptake can cooperate with the disulfidptosis-prone metabolic state of NSCLC, especially in tumors characterized by high SLC7A11 activity, KEAP1/NRF2 dysregulation, or strong glycolytic dependence.

#### 4.2.2. TCM-Derived Compounds Inhibiting Glycolytic Enzymes and Glycolytic Flux in NSCLC

Compared with direct targeting of glucose transporters, current evidence more clearly supports the notion that some TCM-derived compounds suppress NSCLC progression by interfering with key glycolytic enzymes or glycolysis-related metabolic regulators. This is mechanistically relevant to disulfidptosis because the critical metabolic consequences are not only reduced glucose entry but also impaired glycolytic flux and a diminished capacity to generate glucose-derived reducing equivalents under nutrient stress. In NSCLC, several natural compounds have been reported to converge on this metabolic axis. Piperlongumine suppresses NSCLC growth by inhibiting HK2-mediated glycolysis, reducing glucose consumption and lactate production, and disrupting the HK2–VDAC1 interaction [105]. Sinomenine likewise inhibits NSCLC proliferation through downregulation of HK2-mediated aerobic glycolysis, supporting the view that HK2 is a recurrent metabolic target of natural compounds in lung cancer [106]. In a smaller in vitro study, apigenin was also shown to inhibit glycolysis and spheroid formation in H460 cells by reducing HK2 expression [107].

**Figure 3:**
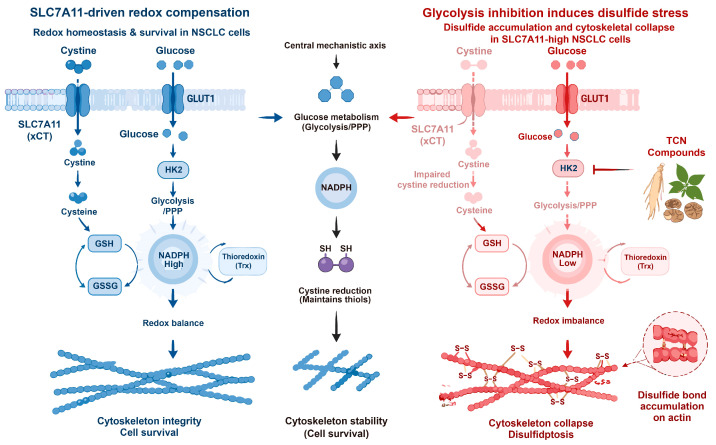
Glucose metabolism-derived NADPH maintains thiol redox homeostasis and prevents disulfide stress-associated cytoskeletal collapse in SLC7A11-high NSCLC cells. Schematic illustration of how SLC7A11-mediated cystine uptake, glucose metabolism, and NADPH production regulate disulfide stress in NSCLC cells. In SLC7A11-high cells, glucose metabolism, particularly glycolysis-associated pentose phosphate pathway activity, supports NADPH generation and cystine-to-cysteine reduction through GSH- and thioredoxin-dependent systems, thereby maintaining thiol homeostasis, actin cytoskeletal integrity, and cell survival. Inhibition of glucose metabolism by TCM-derived compounds may reduce NADPH availability, impair cystine reduction, promote abnormal disulfide bond accumulation in actin cytoskeletal proteins, and increase susceptibility to disulfidptosis-like cytoskeletal collapse under glucose-limited conditions. Faded elements indicate functionally weakened pathways. Blunt-ended lines indicate inhibitory effects.

In addition to HK2-mediated regulation, natural compounds may affect NSCLC glycolysis through downstream enzyme-regulatory pathways. A representative example is β-elemene, which suppresses NSCLC proliferation by targeting the ALDH3A1–HIF-1α/LDHA axis, thereby reducing glycolysis while enhancing oxidative phosphorylation [108]. Taken at the current stage of evidence, these studies support a model in which TCM-derived compounds can restrain NSCLC growth by targeting glycolytic enzymes or glycolysis-associated metabolic programs, particularly at the levels of HK2 and LDHA-related regulation, rather than by being directly validated as inducers of disulfidptosis. Their relevance to disulfidptosis therefore lies mainly in metabolic priming: by weakening glycolytic throughput and reducing metabolic flexibility, these compounds may render SLC7A11-high or otherwise glucose-dependent NSCLC cells less capable of buffering disulfide stress during glucose limitation. This also means that future studies should test more directly whether enzyme-targeting natural compounds can trigger or potentiate bona fide disulfidptosis in metabolically vulnerable NSCLC models. The proposed connection between glucose metabolism, NADPH-dependent cystine reduction, and disulfide stress-associated cytoskeletal collapse in SLC7A11-high NSCLC cells is summarized in Figure 3.

## 5. Discussion

As summarized above, this review focuses on TCM-derived small molecules that target metabolism-associated regulated cell death in NSCLC. Previous reviews have discussed the roles of traditional Chinese medicine, natural products, ferroptosis, or cancer metabolism in tumor therapy. However, many of these reviews have mainly focused on a single cell death modality, the general anticancer effects of natural compounds, or broad metabolic regulation across multiple cancer types. In contrast, the present review integrates compound-level evidence, metabolic vulnerabilities, and regulated cell death pathways within the specific context of NSCLC. This disease-specific and mechanism-oriented framework may help clarify how TCM-derived small molecules could be further developed as metabolism-targeted therapeutic candidates. Based on this perspective, the following sections discuss the major obstacles to clinical translation, emerging technologies for precision medicine, and potential combination strategies.

### 5.1. Bottlenecks and Challenges in Clinical Translation

Although many TCM-derived monomers have demonstrated promising antitumor activity in preclinical studies, their development into first-line clinical therapeutics still faces substantial translational barriers [109]. A central obstacle is the pharmacokinetic gap between the concentrations required to induce regulated cell death in vitro and the drug exposure achievable in vivo [110]. This limitation is not unique to TCM-derived compounds but represents a common challenge in the development of natural products and botanical-derived small molecules. For example, curcumin is limited by poor absorption, rapid metabolism, and rapid systemic elimination; resveratrol shows extensive first-pass metabolism despite relatively efficient intestinal absorption; berberine has low oral exposure partly because of intestinal metabolism and efflux transporter-mediated limitation; and quercetin is constrained by poor aqueous solubility, gastrointestinal instability, low permeability, and extensive metabolism [111,112,113,114,115]. These examples indicate that promising mechanistic activity in cell-based models does not necessarily translate into sufficient plasma or intratumoral exposure [116]. Therefore, future studies of TCM-derived regulated cell death modulators should incorporate pharmacokinetic/pharmacodynamic evaluation, including plasma concentration, tumor accumulation, active metabolites, exposure–response relationships, and safety margins.

Formulation engineering provides a practical route to address these pharmacokinetic barriers. A broad range of delivery systems, including nanocarriers, liposomes, polymeric micelles, solid lipid nanoparticles, nanoemulsions, phytosomes, and cyclodextrin inclusion complexes, have been explored to improve the solubility, stability, circulation time, tissue distribution, and controlled release of poorly soluble natural compounds [117,118]. Representative examples from the broader natural product field support this strategy. Cyclodextrin-based complexes have been used to enhance the apparent solubility and absorption of curcumin; phytosome technology has been reported to improve quercetin exposure; albumin-bound paclitaxel illustrates how reformulation of a natural product-derived anticancer drug can reduce formulation-related limitations and expand clinical utility; and liposomal irinotecan demonstrates that liposomal encapsulation of a natural product-derived agent can prolong systemic circulation and improve therapeutic delivery [119,120,121,122,123,124]. These examples suggest that formulation optimization should be considered an integral part of translational development rather than a secondary technical improvement. However, such systems also introduce new requirements, including characterization of particle size, encapsulation efficiency, release kinetics, storage stability, biodistribution, immunogenicity, scalability, and batch-to-batch reproducibility.

Dose standardization is another major challenge [125,126]. For purified monomers, standardization should include compound purity, stereochemical consistency, impurity profiles, degradation products, residual solvents, and validated analytical methods [127]. For standardized extracts or botanical-derived mixtures, dose definition is more complex because therapeutic activity may depend on multiple constituents acting additively or synergistically [128]. In such cases, dosing should not rely solely on crude extract weight or nominal compound content. Instead, quantitative marker compounds, chemical fingerprints, bioactivity assays, and pharmacokinetic exposure should be integrated to define and compare batches [129]. In the context of regulated cell death-based therapy, functional standardization may be particularly important [15,70,130]. Depending on the proposed mechanism, batch consistency could be evaluated by representative pathway-related readouts, such as GPX4 inhibition, lipid peroxidation, SLC7A11 modulation, copper accumulation, mitochondrial metabolic disruption, DLAT oligomerization, or glycolysis-related metabolic suppression. This approach would help connect chemical quality control with biological activity.

Regulatory issues further complicate the clinical translation of botanical-derived compounds [131,132]. Compared with conventional single-target synthetic drugs, botanical-derived products often have more complex chemical compositions, variable raw materials, and manufacturing processes that may influence therapeutic consistency [133]. Regulatory development therefore requires rigorous documentation of botanical source, plant part, cultivation and harvesting conditions, extraction and processing procedures, chemical characterization, impurity control, stability, and manufacturing reproducibility. If the formulation, extraction process, or source material is changed during development, bridging studies may be required to demonstrate that efficacy and safety remain comparable [134]. For NSCLC applications, future translational studies should combine rigorous chemistry, manufacturing, and control standards with clinically relevant pharmacokinetic/pharmacodynamic modeling, biomarker-guided patient stratification, and systematic toxicity monitoring [135,136,137]. These steps will be essential for moving TCM-derived regulated cell death modulators from mechanistic discovery toward reproducible, clinically testable, and regulatory-compatible therapeutic strategies.

### 5.2. New Technologies and the Outlook for Precision Medicine

To overcome these barriers and maximize therapeutic value, the integration of advanced drug delivery systems with molecular stratification will likely be essential for the modernization of TCM-based oncology therapeutics [138]. Nanodelivery platforms may improve bioavailability and tumor selectivity. Encapsulation of TCM-derived compounds into liposomes, polymeric micelles, nanoparticles, or metal–organic frameworks can increase solubility, prolong circulation time, and increase intratumoral accumulation through passive targeting via enhanced permeability and retention effects or active targeting through ligand engineering. In parallel, emerging proteolysis-targeting chimera (PROTAC) technology offers an additional avenue for precision drug development [139,140]. TCM-inspired PROTAC scaffolds could be engineered to selectively degrade proteins such as GPX4, SLC7A11, or other metabolic dependencies, thereby achieving more potent and specific antitumor activity than conventional free small molecules do. Looking forward, TCM-based treatment strategies for NSCLC may gradually shift from empirical use toward biomarker-guided precision medicine. Patients with elevated SLC7A11 expression or alterations in the expression of KEAP1/NRF2 may preferentially benefit from disulfidptosis-oriented approaches combined with glucose restriction strategies, whereas tumors with high ACSL4 expression and active lipid remodeling may be more responsive to ferroptosis-based interventions. Moreover, because several nonapoptotic death pathways are associated with damage-associated molecular pattern (DAMP) release and immune activation, combining TCM-derived regulated cell death inducers with immune checkpoint blockade may help convert immunologically “cold” tumors into more inflamed, treatment-responsive states. Such combination strategies warrant further mechanistic and clinical investigation.

In this context, a more refined biomarker-based stratification strategy may be important for selecting patients who are most likely to benefit from ferroptosis- or cuproptosis-based therapies [141]. For ferroptosis-oriented interventions, tumors with high SLC7A11, GPX4, FSP1, or NRF2 activity, particularly those harboring KEAP1/NRF2 pathway alterations, may exhibit dependence on antioxidant defense programs and could therefore be vulnerable to agents targeting the system Xc^−^/GSH/GPX4 axis or NRF2-regulated ferroptosis resistance [98,142]. In contrast, increased ACSL4 expression, enhanced polyunsaturated fatty acid-containing phospholipid remodeling, elevated iron uptake, or reduced ferritin-mediated iron storage may indicate a cellular state more permissive to lipid peroxidation and ferroptotic cell death [41,48]. For cuproptosis-based strategies, candidate biomarkers may include FDX1, LIAS, DLAT, LIPT1, and other regulators of mitochondrial protein lipoylation, as well as copper transport-related molecules such as SLC31A1, ATP7A, and ATP7B [70,72]. Tumors with high mitochondrial respiration, active tricarboxylic acid cycle metabolism, and abundant lipoylated mitochondrial proteins may be more sensitive to copper ionophore- or copper metabolism-targeted approaches. However, these biomarkers remain largely investigational, and their predictive value should be validated through multi-omics profiling, functional assays, and prospective clinical studies before routine clinical application [143].

### 5.3. Synergistic Therapeutic Strategies and Future Clinical Applications

Given the growing interest in ferroptosis-based cancer therapy, ferroptosis induction may be particularly valuable when integrated with existing treatment modalities rather than used as a stand-alone strategy [21]. Preclinical evidence suggests that ferroptosis inducers can enhance the antitumor effects of EGFR inhibitors, especially in tumors with adaptive resistance to EGFR-targeted therapy [144,145,146]. This synergy may be related to altered iron metabolism, lipid peroxidation, redox imbalance, and suppression of antioxidant defense pathways such as the system Xc^−^/GSH/GPX4 axis [147]. Therefore, combining ferroptosis induction with EGFR inhibitors may represent a promising approach to overcome drug resistance and improve therapeutic durability.

Ferroptosis-based strategies may also cooperate with immunotherapy and chemotherapy [148]. Ferroptotic tumor cells can release damage-associated molecular patterns and lipid peroxidation products that may reshape the tumor immune microenvironment, potentially increasing responsiveness to immune checkpoint blockade. In addition, several chemotherapeutic agents can increase oxidative stress or disturb intracellular iron and glutathione homeostasis, thereby creating a cellular context that is more permissive to ferroptosis. These findings support the rationale for combining ferroptosis inducers with chemotherapy or immunotherapy in selected tumor types. However, future clinical translation will require careful optimization of drug dosage, treatment sequence, tumor-specific biomarkers, and safety profiles, as excessive lipid peroxidation may also damage normal tissues. Prospective studies are therefore needed to identify patients most likely to benefit from ferroptosis-based combination therapies.

### 5.4. Limitations of This Review

This review has several limitations. First, this article is a narrative review rather than a systematic review or meta-analysis; therefore, although relevant studies were carefully selected, some publications may not have been included. Second, the current evidence for TCM-derived small molecules targeting metabolism-associated cell death in NSCLC remains largely preclinical, and most findings are derived from cell-based or animal studies. Third, direct evidence is relatively strong for ferroptosis, whereas cuproptosis- and disulfidptosis-related mechanisms remain emerging and require further validation in NSCLC-specific models. Finally, the pharmacokinetic properties, bioavailability, toxicity profiles, formulation strategies, and biomarker-guided clinical applicability of many compounds remain insufficiently characterized. Future studies should therefore emphasize standardized experimental designs, in vivo validation, pharmacokinetic and toxicological evaluation, and well-designed clinical investigations.

## 6. Conclusions

TCM-derived small molecules represent a valuable source of bioactive compounds for NSCLC therapy, not merely because of their direct cytotoxic effects but because of their ability to modulate tumor-relevant signaling networks, metabolic vulnerabilities, and regulated cell death pathways. Evidence summarized in this review suggests that these compounds can influence ferroptosis through antioxidant defense, iron metabolism, lipid peroxidation, and the system Xc^−^/GSH/GPX4 axis, while emerging findings also indicate potential links with cuproptosis- and disulfidptosis-related metabolic stress. These mechanisms provide a conceptual framework for understanding how TCM-derived compounds may complement existing targeted therapy, chemotherapy, and immunotherapy, particularly in tumors characterized by redox imbalance, metabolic plasticity, or treatment resistance. Nevertheless, clinical translation remains limited by insufficient mechanistic validation, poor bioavailability, lack of standardized formulations, incomplete biomarker stratification, and limited evidence from clinically relevant models. Future studies should therefore move beyond single-compound and single-pathway paradigms toward multi-target therapeutic strategies integrated with precision oncology. Biomarker-guided patient selection, rational nanodelivery systems, combination regimens, and targeted protein degradation approaches such as PROTAC-inspired strategies may improve specificity and therapeutic efficacy. Importantly, these strategies should be validated in patient-derived xenografts, genetically engineered mouse models, orthotopic NSCLC models, and immune-competent systems to better capture tumor heterogeneity, pharmacological behavior, toxicity, and immune microenvironmental effects. Overall, TCM-derived small molecules offer promising leads for developing metabolism-centered and regulated cell death-based therapeutic strategies for NSCLC, but their future application will require rigorous mechanistic studies, optimized delivery platforms, clinically relevant preclinical validation, and well-designed translational research.

## Figures and Tables

**Table 1:** TCM-derived small molecules targeting metabolism-associated cell death pathways in NSCLC.

	TCM-Derived Small Molecules	Structure	Cell Death Modality		TCM-Derived Small Molecules	Structure	Cell Death Modality
1	β-Elemene	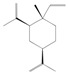	Ferroptosis; Potential disulfidptosis priming	10	Polyphyllin III	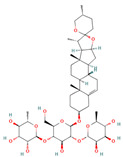	Ferroptosis-related sensitization
2	Periplocin	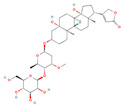	Ferroptosis	11	Betulin	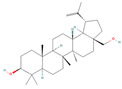	Ferroptosis/drug-resistance reversal
3	Ophiopogonin B	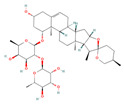	Ferroptosis	12	Celastrol	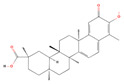	Cuproptosis-associated copper-dependent cell death
4	Dihydroartemisinin, DHA	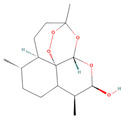	Ferroptosis/mixed regulated cell death	13	Curcumin	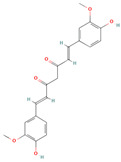	Copper-homeostasis modulation
5	Artesunate	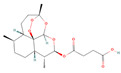	Ferroptosis/mixed regulated cell death	14	Triptolide	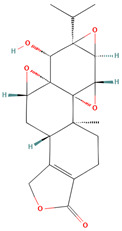	Cuproptosis-related vulnerability
6	Cucurbitacin B	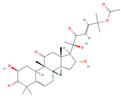	Ferroptosis	15	Gambogic acid	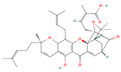	Cuproptosis-related proteotoxic vulnerability
7	Shikonin	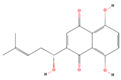	Ferroptosis-related cell death	16	Piperlongumine	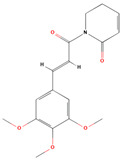	Potential disulfidptosis priming
8	Brusatol	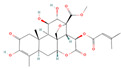	Ferroptosis-related sensitization	17	Sinomenine	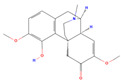	Potential disulfidptosis priming
9	Trigonelline	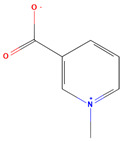	Ferroptosis-related sensitization	18	Apigenin	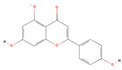	Potential disulfidptosis priming

Carbon atoms and bonds are shown in black, oxygen atoms in red, and hydrogen atoms in blue-gray. The numbers are used solely as sequential identifiers for the compounds and do not indicate ranking, priority, or evidence strength.

## Data Availability

No new data were created or analyzed in this study. Data sharing is not applicable to this article.

## Supplementary Materials

The following supporting information can be downloaded at https://www.mdpi.com/article/10.3390/ph19071026/s1. Table S1: TCM-derived small molecules targeting metabolism-associated cell death pathways and related vulnerabilities in NSCLC.

## Abbreviations

The following abbreviations are used in this manuscript:

ACSL4	Long-chain acyl-CoA synthetase 4
ALDH3A1	Aldehyde dehydrogenase 3 family member A1
α-KGDH	Alpha-ketoglutarate dehydrogenase
ATF3	Activating transcription factor 3
ATOX1	Antioxidant 1 copper chaperone
ATP	Adenosine triphosphate
ATP7A	ATPase copper transporting alpha
ATP7B	ATPase copper transporting beta
AURKA	Aurora kinase A
BCS	Biopharmaceutics Classification System
BH4	Tetrahydrobiopterin
CoA	Coenzyme A
CoQ	Coenzyme Q
CoQ10	Coenzyme Q10
CoQ10H2	Ubiquinol
COX	Cytochrome c oxidase
CTR1	Copper transporter 1
DAMP	Damage-associated molecular pattern
DHA	Dihydroartemisinin
DHODH	Dihydroorotate dehydrogenase
DLAT	Dihydrolipoamide S-acetyltransferase
EGFR	Epidermal growth factor receptor
ERK	Extracellular signal-regulated kinase
F-actin	Filamentous actin
FDX1	Ferredoxin 1
Fe-S	Iron–sulfur
FSP1	Ferroptosis suppressor protein 1
GCH1	GTP cyclohydrolase 1
GCLM	Glutamate-cysteine ligase modifier subunit
GLUT	Glucose transporter
GLUT1	Glucose transporter 1
GPX4	Glutathione peroxidase 4
GSH	Reduced glutathione
GSSG	Oxidized glutathione
HIF-1α	Hypoxia-inducible factor 1 alpha
HK2	Hexokinase 2
HSP70	Heat shock protein 70
HSP90	Heat shock protein 90
ICIs	Immune checkpoint inhibitors
KEAP1	Kelch-like ECH-associated protein 1
LDHA	Lactate dehydrogenase A
LIAS	Lipoic acid synthetase
LIP	Labile iron pool
LPCAT3	Lysophosphatidylcholine acyltransferase 3
MAPK	Mitogen-activated protein kinase
NAD(P)H	Reduced nicotinamide adenine dinucleotide phosphate/nicotinamide adenine dinucleotide
NADPH	Reduced nicotinamide adenine dinucleotide phosphate
NCOA4	Nuclear receptor coactivator 4
NRF2	Nuclear factor erythroid 2-related factor 2
NSCLC	Non-small cell lung cancer
PDH	Pyruvate dehydrogenase
PE	Phosphatidylethanolamine
PE-PUFAs	Polyunsaturated fatty acid-containing phosphatidylethanolamines
PL-OOH	Phospholipid hydroperoxide
POLD1	DNA polymerase delta 1
PPP	Pentose phosphate pathway
PROTAC	Proteolysis-targeting chimera
PUFA	Polyunsaturated fatty acid
PUFA-CoA	Polyunsaturated fatty acyl-coenzyme A
RCD	Regulated cell death
ROS	Reactive oxygen species
SDHB	Succinate dehydrogenase complex iron–sulfur subunit B
SIRT3	Sirtuin 3
SLC31A1	Solute carrier family 31 member 1
SLC3A2	Solute carrier family 3 member 2
SLC7A11	Solute carrier family 7 member 11
SOD1	Superoxide dismutase 1
SRF	Serum response factor
STAT3	Signal transducer and activator of transcription 3
System Xc^−^	Cystine/glutamate antiporter system
TCA	Tricarboxylic acid
TCM	Traditional Chinese medicine
TFEB	Transcription factor EB
TFR1	Transferrin receptor 1
VDAC1	Voltage-dependent anion channel 1
WAVE	WASP-family verprolin-homologous regulatory complex
xCT	Cystine/glutamate transporter
