# Supplementary material for: TCM-Derived Small Molecules Targeting Metabolic Vulnerabilities in NSCLC: Ferroptosis-Centered Mechanisms and Emerging Cuproptosis- and Disulfidptosis-Related Vulnerabilities
Source: Pharmaceuticals (Basel). 2026 Jun 30;19(7):1026. doi: 10.3390/ph19071026 (PMC13415089; doi:10.3390/ph19071026)
Supplement: Supplementary file 1 [file pharmaceuticals-19-01026-s001.zip › pharmaceuticals-4369844-supplementary.pdf]

Table S1. TCM-derived small molecules targeting metabolism-associated cell death pathways and related vulnerabilities in NSCLC

Cell death modality	TCM-derived compound(s)	Metabolic vulnerability / target axis	Mechanistic summary in NSCLC	Evidence status and therapeutic relevance	References
Ferroptosis	β-Elemene	SLC7A11-GSH-GPX4 antioxidant defense; GPX4 turnover	Promotes TFEB-mediated lysosomal degradation of GPX4, impairing lipid-peroxide detoxification and inducing ferroptotic cell death. It has also been linked to enhanced EGFR-TKI sensitivity through ferroptosis-related mechanisms.	Direct NSCLC evidence; one of the better-characterized TCM-derived ferroptosis inducers in NSCLC. Potentially relevant to therapy-resistant and EGFR-targeted settings.	[49,50]
Ferroptosis	Periplocin	NRF2-dependent antioxidant program upstream of SLC7A11/GPX4	Induces NRF2 degradation, thereby weakening NRF2-driven redox defenses and potentiating ferroptotic cell death.	Direct NSCLC evidence; Relevant to NRF2-driven ferroptosis resistance, although further in vivo and translational validation is still required.	[51]
Ferroptosis	Ophiopogonin B	Ferroptosis-related gene regulation; AURKA-associated signature	Regulates an AURKA-associated ferroptosis-related gene signature and alters ferroptosis markers, including MDA, GSH, intracellular iron, and mitochondrial membrane potential.	Direct NSCLC evidence, but mechanistic depth is less comprehensive than that for β-elemene and periplocin.	[52]
Ferroptosis / mixed RCD	Artemisinin derivatives, including DHA and artesunate	Iron homeostasis, labile iron pool, ROS generation	The iron-reactive endoperoxide bridge can be activated by intracellular iron to generate radical species, intensifying oxidative injury and apoptosis/ferroptosis-associated death. Ferritinophagy/free-iron mechanisms remain incompletely validated in NSCLC.	Direct NSCLC antitumor evidence with mixed apoptosis/ferroptosis features; some mechanistic branches are extrapolated from broader cancer models.	[54-56]
Ferroptosis	Cucurbitacin B	STAT3 signaling; oxidative stress; iron-dependent lipid peroxidation	Targets STAT3 and promotes ferroptosis-associated oxidative stress, linking oncogenic signaling inhibition with ferroptotic vulnerability.	Direct NSCLC evidence; potential option for STAT3-activated tumors.	[57]
Ferroptosis-related, non-NSCLC evidence	Shikonin	ATF3-mediated ROS accumulation and ferroptosis-related signaling	Suppresses lung cancer growth through ATF3-dependent ferroptosis and ROS accumulation; however, the strongest direct evidence cited in the review concerns small cell lung cancer.	Evidence mainly comes from SCLC rather than NSCLC; included only as supportive lung cancer evidence and should not be used to support NSCLC-specific conclusions.	[58]
Ferroptosis-related sensitization	Brusatol	NRF2/KEAP1 antioxidant signaling	Promotes NRF2 ubiquitination and proteasomal degradation, suppressing NRF2-dependent antioxidant genes and potentially lowering the threshold for ferroptosis.	NSCLC evidence for NRF2 inhibition and chemosensitization; ferroptosis-specific validation remains insufficient.	[61]
Ferroptosis-related sensitization	Trigonelline	NRF2 nuclear translocation; GSH synthesis	Blocks NRF2 nuclear translocation and downregulates antioxidant genes such as GCLM, reducing glutathione synthesis and favoring lipid peroxidation.	Indirect evidence; canonical ferroptosis induction in NSCLC requires further validation.	[62]
Ferroptosis-related sensitization	Polyphyllin III	Lipid remodeling; ACSL4-mediated PUFA-phospholipid peroxidation	In breast cancer models, polyphyllin III induces ACSL4-mediated lipid peroxidation and ferroptosis. Its relevance to NSCLC remains extrapolative and requires direct validation.	Non-NSCLC supportive evidence; should be framed only as a ferroptosis-related lipid-remodeling example, not as an NSCLC-validated compound.	[65,66]
Ferroptosis / drug-resistance reversal	Betulin plus gefitinib	Ferroptosis-associated EGFR-TKI sensitization	Combined treatment induces ferroptosis and improves antitumor responses in EGFR wild-type/KRAS-mutant NSCLC models.	Direct NSCLC combination evidence; supports ferroptosis activation as a resistance-reversal strategy.	[69]
Cuproptosis-associated copper-dependent cell	Celastrol	SRF-SLC31A1/CTR1-mediated copper uptake; FDX1-DLAT-Fe-S cluster axis	Upregulates SLC31A1/CTR1 through SRF-dependent transcriptional control, causing copper accumulation. Associated changes include GSH depletion, DLAT oligomerization, HSP70	Direct NSCLC evidence; the most convincing cuproptosis-associated TCM-derived compound in the review.	[90,91]

Cell death modality	TCM-derived compound(s)	Metabolic vulnerability / target axis	Mechanistic summary in NSCLC	Evidence status and therapeutic relevance	References
death			induction, loss of Fe-S cluster-related proteins, mitochondrial depolarization and ATP depletion.		
Copper-homeostasis modulation	Curcumin	ATOX1-mediated copper trafficking	Binds the copper chaperone ATOX1, suppresses intracellular copper accumulation and inhibits ATOX1-mediated copper signaling.	Direct copper-homeostasis evidence in NSCLC, but not direct cuproptosis induction. Better framed as copper-transport modulation.	[92]
Cuproptosis-related vulnerability	Triptolide	Mitochondrial bioenergetics; SIRT3; respiratory chain complexes I/II	Impairs mitochondrial function in a p53-dependent manner partly through SIRT3 dysregulation and reduced respiratory complex I/II activity, potentially altering the metabolic background that determines copper sensitivity.	Indirect evidence; cuproptosis was not directly examined. Note toxicity/therapeutic-window limitations when discussing translation.	[93]
Cuproptosis-related proteotoxic vulnerability	Gambogic acid	HSP90-centered proteostasis network	Inhibits HSP90 and may weaken protein quality-control capacity. Because cuproptosis involves copper-induced protein aggregation and proteotoxic stress, HSP90 inhibition may lower the threshold for copper-triggered injury.	Indirect evidence; requires direct validation as a cuproptosis sensitizer in NSCLC.	[94,95]
Potential disulfidptosis priming	Piperlongumine	HK2-mediated glycolysis; HK2-VDAC1 interaction	Suppresses HK2-mediated glycolysis, reduces glucose consumption and lactate production, and disrupts HK2-VDAC1 interaction. Reduced glycolytic flux may limit NADPH availability and enhance disulfide stress in SLC7A11-high NSCLC cells.	Indirect/proposed disulfidptosis relevance; direct disulfidptosis has not been validated.	[105]
Potential disulfidptosis priming	Sinomenine	HK2-mediated aerobic glycolysis	Downregulates HK2-mediated glycolysis and inhibits NSCLC proliferation, potentially reducing glucose-derived reducing equivalents required to buffer disulfide stress.	Indirect/proposed relevance; candidate metabolic sensitizer for glucose-dependent or SLC7A11-high NSCLC.	[106]
Potential disulfidptosis priming	Apigenin	HK2 expression and glycolytic activity	Reduces HK2 expression, inhibits glycolysis and suppresses spheroid formation in H460 cells, potentially creating a redox state permissive for disulfide stress under glucose limitation.	Indirect/proposed evidence based on a smaller in vitro study.	[107]
Potential disulfidptosis priming	β-Elemene	ALDH3A1-HIF-1α/LDHA glycolytic axis	Suppresses glycolysis by targeting the ALDH3A1-HIF-1α/LDHA axis and enhances oxidative phosphorylation, potentially reducing glycolytic flexibility and disulfide-stress-buffering capacity.	Indirect/proposed disulfidptosis relevance; direct disulfidptosis requires validation.	[108]

Note： Direct evidence indicates experimental validation of the indicated death modality in NSCLC models using pathway-relevant markers and, where available, rescue experiments. Indirect or proposed evidence indicates modulation of a mechanistically relevant pathway without full confirmation of bona fide cell death by pathway-specific rescue assays and canonical molecular features.
